# Heterologous prime-pull mucosal vaccination with an adjuvanted RBD vaccine elicits robust IgA production and protects against SARS-CoV-2

**DOI:** 10.3389/fimmu.2025.1673460

**Published:** 2025-09-19

**Authors:** Allyson H. Hirsch, Calder R. Ellsworth, William A. Lewis, Ryan Craig, Amy E. Meyer, Jonatan Maldonado, Frania Ramirez Lopez, Syamala Rani Thimmiraju, James B. McLachlan, Xuebin Qin, Nicholas J. Maness, Jeroen Pollet, Ulrich Strych, Maria Elena Bottazzi, Peter J. Hotez, Lisa A. Morici

**Affiliations:** ^1^ Department of Microbiology and Immunology, Tulane University School of Medicine, New Orleans, LA, United States; ^2^ Tulane National Primate Research Center, Covington, LA, United States; ^3^ Department of Pathology and Laboratory Medicine, Tulane University School of Medicine, New Orleans, LA, United States; ^4^ Texas Children’s Hospital Center for Vaccine Development, Departments of Pediatrics and Molecular Virology and Microbiology, Baylor College of Medicine, Houston, TX, United States; ^5^ National School of Tropical Medicine, Baylor College of Medicine, Houston, TX, United States; ^6^ Department of Biology, Baylor University, Waco, TX, United States

**Keywords:** intranasal, vaccine, COVID-19, mucosal immunity, adjuvant

## Abstract

Despite the efficacy of approved severe acute respiratory syndrome coronavirus 2 (SARS-CoV-2) vaccines in preventing severe disease and death, breakthrough infections continue to occur in vaccinated individuals, contributing to further viral mutation and spread. These limitations may be attributable to the poor induction of mucosal immunity by parenteral vaccination. Mucosal adjuvants, such as T-vant, can enhance vaccine-induced immune responses through the generation of antigen-specific antibodies and T cells in the respiratory tract. In this study, we evaluated the protective efficacy of adjuvanted SARS-CoV-2 receptor binding domain (RBD) subunit vaccines administered by homologous and heterologous routes. Immunized mice were challenged with SARS-CoV-2-XBB.1.5 and monitored for weight loss and survival. Lung and nasopharynx tissues were collected at pre-scheduled timepoints to assess viral loads and histopathology. Additionally, vaccine-induced humoral and cell-mediated immune responses were evaluated in the mucosal and systemic compartments. A prime-pull vaccination strategy – comprising an intramuscular prime immunization with aluminum hydroxide (alum) and CpG-adjuvanted RBD followed by an intranasal boost with T-vant-adjuvanted RBD – conferred protection against mortality and lung pathology and cleared virus from the nasopharynx by three days post infection. The prime-pull vaccine regimen elicited superior anti-RBD IgA in the bronchoalveolar lavage fluid and nasal washes, when compared to other vaccine groups. Given that much of the global population has already received parenteral SARS-CoV-2 vaccination or has been naturally exposed, a prime-pull approach could leverage pre-existing systemic immunity using a single mucosal boost.

## Introduction

The coronavirus disease of 2019 (COVID-19) pandemic generated an unprecedented global effort to develop effective vaccines against severe acute respiratory syndrome coronavirus 2 (SARS-CoV-2). This urgency led to the rapid development of vaccines utilizing replication-deficient adenoviral vectors (1) and lipid nanoparticle-encapsulated mRNA technology (2, 3) – both of which demonstrated remarkable efficacy in preventing severe disease and mortality, and significantly reducing infection-related complications. In the United States (US), approved SARS-CoV-2 vaccines are administered intramuscularly and elicit robust systemic immunity, characterized by high titers of circulating antibodies, memory B cells, and effector and memory T cells (4–6).

Despite initial protective efficacy exceeding 90% against symptomatic disease, SARS CoV-2 mRNA vaccine-induced immunity waned over time (7, 8). Neutralizing antibody titers declined by up to 50% within six months post-mRNA vaccination (9), necessitating frequent booster doses to maintain protection. Additionally, breakthrough infections occurred, particularly with highly immune-evasive variants such as Omicron (10). Beyond antigenic variation, these breakthrough infections may be attributed to a documented lack of mucosal immunity generated following intramuscular immunization with SARS-CoV-2 vaccines (11, 12). Since SARS-CoV-2 enters through the respiratory tract, vaccines that fail to elicit mucosal immunity may be less effective at preventing infection and viral transmission. Thus, next-generation vaccines that aim to induce robust mucosal immunity may better clear infection and curb viral spread, while also mitigating further viral evolution.

Mucosal immunity plays a crucial role in preventing respiratory infections by combatting pathogens at their entry site (13). Secretory IgA, the predominant antibody at mucosal tissues, has been shown to limit viral replication and prevent systemic dissemination of SARS-CoV-2 in mice and humans (14). Secretory IgA may also limit virus shedding and therefore transmission (15). Additionally, lung-resident T and B cells improve cross-protection against Omicron sub-lineages (16–18). Given these advantages, mucosal vaccination represents a promising strategy for next-generation SARS-CoV-2 vaccines (19), as mucosal immunization drives localized immune responses (20, 21). However, only two mucosal COVID-19 vaccines have been approved worldwide (22). These include India’s iNCOVACC, an intranasal chimpanzee adenoviral 36 (ChAd36)-vectored SARS-CoV-2 Spike vaccine (23), and China’s Convidecia Air, an inhaled adenoviral 5 (Ad5)-vectored aerosol vaccine (24). Both rely on adenovirus viral vectors that are no longer recommended for prevention of SARS CoV-2 in the US due to rare thrombotic events (25). Most individuals globally have already received intramuscular SARS-CoV-2 vaccines or have been exposed to the virus naturally. Therefore, heterologous prime-pull immunization, whereby a prime immunization/exposure is followed by a mucosal boost, may constitute a favorable approach to enhance mucosal protection. In this immunization scenario, intramuscular injection or “prime” establishes robust systemic immunity, while the intranasal “pull” recruits immune cells to the respiratory tract for rapid protection upon infection (26).

A receptor binding domain (RBD)-based subunit vaccine has shown superior protection against SARS CoV-2 in mice. The vaccine is adjuvated with aluminum hydroxide (alum) and CpG oligodeoxynucleotide (CpG ODN; collectively AH: CpG) and given in two doses intramuscularly (27–29). Alum and CpG adjuvants are well-established for use in parenteral vaccines, where they promote strong systemic humoral and cellular immunity (30–32). In humans, such RBD-based subunit vaccines with AH: CpG for COVID-19 were administered to an estimated 100 million people in India and Indonesia as Corbevax and IndoVac, respectively (33). However, this combination adjuvant cannot be administered mucosally (34, 35). T-vant is a novel outer membrane vesicle (OMV)-based adjuvant that can be delivered mucosally to protect against respiratory (36) and enteric pathogens (37). We hypothesized that a heterologous prime-pull vaccination, using RBD + AH: CpG intramuscularly followed by RBD + T-vant intranasally, would induce robust systemic and mucosal immunity, resulting in superior protection against mortality, viral colonization, and tissue pathology in the respiratory tract. The immunogenicity and protective efficacy of this approach is compared to homologous prime-boost immunization with RBD +AH: CpG, RBD + T-vant, and mRNA vaccines.

## Materials and methods

### Ethics statement

This study was performed in strict accordance with the Guide for the Care and Use of Laboratory Animals of the National Institutes of Health (NIH). The protocols were approved by the Tulane University Institutional Animal Care and Use Committee (IACUC; Protocol number 1831). Tulane University School of Medicine is fully accredited by the Association for the Assessment and Accreditation of Laboratory Animal Care-International (AAALAC).

### Viral strains and growth conditions

For the SARS-CoV-2 Omicron subvariant XBB 1.5, the isolate was obtained as a seed stock from BEI Resources (NR-59105) and expanded on VeroE6-TMPRSS2 cells in Dulbecco’s Modified Eagle Medium (DMEM) with 2% fetal bovine serum (FBS). The final viral stock was tittered by plaque and 50% tissue culture infectious dose (TCID50) assays and deep sequenced to ensure sequence homology with original isolate.

### Mice

Male and female K18-hACE2 transgenic mice (C57Bl/6 background; strain B6.Cg-Tg(K18-ACE2)2Prlmn/J; 034860) and wild-type C57Bl/6J mice (000664) were purchased from The Jackson Laboratory (Bar Harbor, ME) and housed under pathogen-free conditions at Tulane University. For immunology assays, 6- to 8-week-old C57Bl/6J mice were used. For SARS-CoV-2-XBB.1.5 challenge experiments, 6- to 8-week-old or 20- to 28-week-old K18-hACE2 mice were utilized. Sample sizes were determined based on prior studies with comparable experimental designs to ensure adequate statistical power. Age- and sex-matched mice were randomly assigned to experimental groups prior to the initiation of experimental procedures. Mice were housed in groups of up to 5 animals per cage under a 12-hour light/dark cycle with *ad libitum* access to rodent chow and water.

### Vaccination

The adjuvants and their doses used were: Alhydrogel adjuvant 2% (200 µg; InvivoGen, San Diego, CA), T-vant (0.5 µg intramuscular (IM) or 5 µg intranasal (IN); *T-vant has its own record as VO_0005375 linked:* *https://vac.niaid.nih.gov/view?id=61* ), and CpG-ODN 55.2 (10 µg; Vaxine, Adelaide, South Australia; *CpG-55.2 has its own record as VO_0006094 linked:* *https://vac.niaid.nih.gov/view?id=10* ). The antigen and its dose used was: XBB.1.5 RBD (7 µg IM and IN). Recombinant RBD constructs were generated by Baylor College of Medicine (27–29). The Omicron XBB.1.5 subvariant mRNA vaccine (Comirnaty; BioNTech-Pfizer, New York, NY) was obtained from the Tulane University pharmacy. Stock solutions (100 µg/mL) were diluted 1:3 in phosphate-buffered saline (PBS) to administer a final dose of 1.67 µg per mouse (27). Control groups included antigen-only, adjuvant-only, and naïve (unimmunized) mice to account for non-specific immune effects.

For intranasal vaccination, mice received a total volume of ≤10 µL, divided equally between both nostrils. Intramuscular vaccinations were delivered as 50 µL injections into the caudal thigh. Prior to immunization, all mice were briefly anesthetized with isoflurane. Prime-boost vaccination schedules and necropsy timepoints varied across experiments, as detailed in the figure legends. Each experimental group contained equal numbers of male and female mice.

### T-vant adjuvant

The T-vant used in this study was GLP-grade material manufactured by Catalent Biologics under NIH Adjuvant Development contract number 272201800045 C as described previously (36). The final T-vant formulation contains 100 mM Tris Base, 3% Sucrose, 110 mM NaCl, pH 7.4, with a protein concentration of 0.5 mg/mL and final endotoxin measurements of 14 EU/mL.

### Challenge studies

K18-hACE2 mice were anesthetized with isoflurane gas and inoculated intranasally 30 days post-boost with 1.7x10^5^ plaque forming units (PFU) of SARS-CoV-2-XBB.1.5 in a total volume of 50 µL (25 µL per nare). This challenge dose is lethal to aged mice (>26 week) so 26- to 28-week-old mice were used for survival studies. For determination of viral burdens and pathology, 6- to 8-week-old mice were used as the same dose of 1.7 x 10^5^ PFU XBB.1.5 is sub-lethal in young mice. Mice were monitored daily for clinical signs and weighed for 14 days post-infection (DPI) to assess disease progression. To quantify viral burden, mice were euthanized via carbon dioxide (CO_2_) asphyxiation at days 0 (2 naïve mice), 1, 3, 7, and 14 with 3 mice sacrificed per timepoint per vaccine group. Lungs and nasopharynx tissues were collected and stored in TRIzol reagent (Invitrogen, Waltham, MA) to preserve RNA integrity for viral load quantification. To assess tissue histopathology, lungs (n=3 per vaccine group) were inflated with and immersed in Z-FIX fixative (Anatech Ltd, Battle Creek, MI) 4 DPI and processed for pathological evaluation.

### Viral load quantification

Quantitative PCR with reverse transcription (RT-qPCR) was conducted following established protocols (38). Briefly, RNA was isolated from collected lung and nasopharynx samples using the RNeasy Plus Mini Kit (Qiagen, Hilden, Germany). Subgenomic (sg) RNA encoding the nucleocapsid (N) protein was amplified using the cycling conditions developed and shared by D. Hartigan-O’Connor and J. Dutra (U. California-Davis). Primers and probes sequences for the N protein were as follows: forward 5′-CGATCTCTTGTAGATCTGTTCTC-3′, reverse 5′-GGTGAACCAAGACGCAGTAT-3′, probe 5′-FAM-TAACCAGAATGGAGAACGCAGTGGG-BHQ1-3′. Reactions were prepared in 20 µL volumes containing 5 µL RNA template, 900 mM primers, 250 nM probe, and TaqPath 1-Step RT-qPCR master mix, CG (Thermo Fisher Scientific, Waltham, MA). Thermal cycling parameters included an initial uracil *N*-glycosylase (UNG) incubation at 25 °C for 2 min, reverse transcription at 50 °C for 15 min, and Taq polymerase activation at 95 °C for 2 min, followed by 40 cycles of denaturation at 95 °C for 3 sec and annealing/extension at 60 °C for 30 sec. An RNA standard curve was included to quantify viral copy numbers, expressed as log_10_(copies/µL).

### Lung histology

Pulmonary inflammation was assessed in immunized and naïve mice by histopathological analysis of lung tissues were collected 4 DPI. Following euthanasia, a 22-gauge catheter was inserted into the trachea, and lungs were inflated with 2 mL of Z-FIX fixative (Anatech LTD, Battle Creek, MI) administered via a 3 mL syringe. Tissues were fixed by immersion in Z-FIX at room temperature and subsequently transferred to the Tulane University Health Sciences Center Pathology Core Laboratory for further processing. Fixed lungs were embedded in paraffin, sectioned into 5 µm thickness, and stained with hematoxylin and eosin (H&E) using standard protocols. Whole-lung sections were systematically scanned at 2x low-power and 20x high-power magnification to assess global and localized inflammatory pathology; representative images were captured using a digital camera (Nikon Eclipse Ei, Nikon Healthcare, Tokyo, Japan). For quantification of percent area of inflammation, stained tissue sections were scanned using a Zeiss Axio Scan.Z1 and whole slide images were viewed and captured using HALO image analysis software (HALO v3.2, Indica Labs, Albuquerque, NM) (39).

### *Ex vivo* restimulation assay and intracellular cytokine staining

Mice were euthanized via CO_2_ asphyxiation, after which lung and spleen tissues were harvested and placed in sterile 1.5 mL tubes containing 700 µL sterile PBS and placed on ice until processing. Lung tissues were enzymatically digested in a solution of 2 mg/mL collagenase IV (Sigma Aldrich, St. Louis, MO) and 20 U/mL DNase I (Sigma Aldrich, St. Louis, MO) in media, followed by agitation at 233 rmp for 1 hour at 37 °C. Mechanical dissociation of lung and spleen tissues was performed using a 70 µm cell strainer (Fisher Scientific, Hampton, NH) and the rubber plunger of a syringe to generate single-cell suspensions. Cells were resuspended in Iscove’s Modified Dulbecco’s Medium (IMDM) supplemented with GlutaMAX (Gibco, Waltham, MA) and 10% FBS. Red blood cells were lysed using Ammonium-Chloride-Potassium (ACK) Lysing Buffer (Gibco, Waltham, MA) for 3 minutes at room temperature (RT). Viable cells were counted with Trypan Blue (Gibco, Waltham, MA), and 1.5x10^6^ live cells were seeded into 96-well culture-treated plates (Fisher Scientific, Hampton, NH). Cells were stimulated with 1 µg/mL RBD protein (generated by Baylor College of Medicine) for 20 hours at 37 °C under 5% CO_2_. GolgiStop (BD Biosciences, Franklin Lakes, NJ) was added 4 hours prior to the end of incubation. Positive control wells were treated with 60 ng/mL phorbol 12-myristate 13-acetate (PMA; ThermoFisher Scientific, Waltham, MA) and 1 µg/mL ionomycin (ThermoFisher Scientific, Waltham, MA). Negative controls included unstimulated cells and cells treated with 1 µg/mL ovalbumin.

For flow cytometric analysis, cells were stained with Zombie NIR Fixable Viability Dye (BioLegend, San Diego, CA) to distinguish live/dead populations, followed by surface staining with anti-mouse fluorophore-conjugated antibodies: CD45:FITC (clone I3/2.3), CD11b:PerCP-Cyanine5.5 (clone M1/70), CD19:PerCP-Cyanine5.5 (clone 6D5), F4/80:PerCP-Cyanine5.5 (clone BM8), Gr-1:PerCP-Cyanine5.5 (clone RB6-8C5), CD8:Pacific Blue (clone 53-6.7), CD4:Brilliant Violet 510 (clone RM4-5), CD44:PE-Dazzle 594 (clone IM7), and CD3:PE-Cyanine7 (clone 17A2). Cells were fixed and permeabilized using the BD Cytofix/Cytoperm Kit (BD Biosciences, Franklin Lakes, NJ) and intracellularly stained with IFN-γ:APC (clone XMG1.2), IL-4:Brilliant Violet 605 (clone 11B11), and IL-17:PE (clone TC11-18H10.1). All antibodies were sourced from BioLegend (San Diego, CA). Stained cells were analyzed on a BD LSRFortessa flow cytometer (BD Biosciences, San Jose, CA) and data were analyzed using FloJo v10.10 software (BD Biosciences, San Jose, CA).

### Indirect ELISA

Blood samples were collected from immunized mice at two weeks post-boost for endpoint antibody titers. Terminal bleeds (~600 µL) were performed via cardiac puncture following euthanasia. Blood was transferred to microtainer SST tubes (BD, Franklin Lakes, NJ) to isolate serum. Bronchoalveolar lavage fluid (BALF) was collected by canulating the trachea of mice, instilling 1 mL of sterile PBS into the lungs, then retrieved the fluid into a syringe. Nasal washes (NW) were collected by canulating the trachea of mice (directed towards the nose) and slowly lavaging with 500 µL of sterile PBS. Blood, BALF, and NW samples were stored at -80 °C until further processing.

For ELISA, 96-well plates were coated with 250 ng/well RBD. Serially diluted serum, BALF, or NW samples were incubated in coated plates, followed by horse radish peroxidase (HRP)-conjugated goat anti-mouse IgG (Invitrogen, Waltham, MA) or IgA (ThermoFisher Scientific, Waltham, MA) secondary antibodies. Plates were washed three times with PBS containing 0.05% Tween-20 (PBS-T) between steps. Bound antibodies were detected using a 3,3’,5,5’-Tetramethylbenzidine (TMB) peroxidase substrate kit (SeraCare, Milford, MA), and reactions were quenched with stop solution when the highest-concentrated wells 2.0-2.5 absorbance units. Absorbance was measured at 450 nm with a microplate reader within 30 min of stopping. Endpoint titers were defined as the highest serum dilution exceeding the cutoff value (mean absorbance of blanks + 3*standard deviation).

### Pseudovirus assay

Replication-incompetent lentiviral vectors pseudotyped with SARS-CoV-2 spike protein variant (XBB.1.5) were generated as previously described (29). These pseudoviruses incorporated a luciferase reporter system to enable quantification of viral entry into cells (40). Neutralization assays were performed using human 293 T-hACE2 cells, cultured under standard *in vitro* conditions. For neutralization testing, heat-inactivated serum samples were serially diluted and incubated with 10 µL of pseudovirus for 1 hour at 37 °C. The serum-pseudovirus mixture (100 µL) were then transferred to 293 T-hACE2 cells in 96-well culture plates. Following a 48-hour incubation at 37 °C with 5% CO_2_, cells were lysed with 100 µL of Glo Lysis buffer for 15 mins at RT. Lysates (50 µL) were combined with 50 µL of luc substrate (Promega Luciferase Assay System, Madison, WI), luminescent signal (relative luminescence units, RLUs) was quantified using a Luminometer (Biosynergy H4, Elk Grove Village, IL). Neutralization potency was determined by calculating the 50% inhibitory dilution (IC50), defined as the serum dilution required to reduce pseudovirus infection by 50% relative to negative controls.

### Statistical analysis

Statistical analyses were performed using GraphPad Prism software (version 10.4.1; GraphPad Software, San Diego, CA). Data are presented as mean ± standard error of the mean (s.e.m.). For challenge studies, percent weight loss was analyzed using a modified Chi-squared method corrected for deviation from normality due to small to moderate sample sizes, as outlined in ref (41). Survival outcomes were evaluated with the Mantel-Cox log-rank test. T cell and antibody response data were compared across experimental groups using a one-way ANOVA with a *post hoc* multiple comparisons test (Tukey’s and Holm-Sidak’s post-tests; specified in figure legends). Significance thresholds were defined as follows: ^ns^ *p > 0.05*, *p ≤ 0.05, **p ≤ 0.01, ***p ≤ 0.001, or ****p ≤ 0.0001. In figures, significance bars denote pairwise comparisons between groups achieving significance.

## Results

### Immunization route impacts protective efficacy of adjuvanted RBD subunit vaccines in aged mice

**Figure 1:**
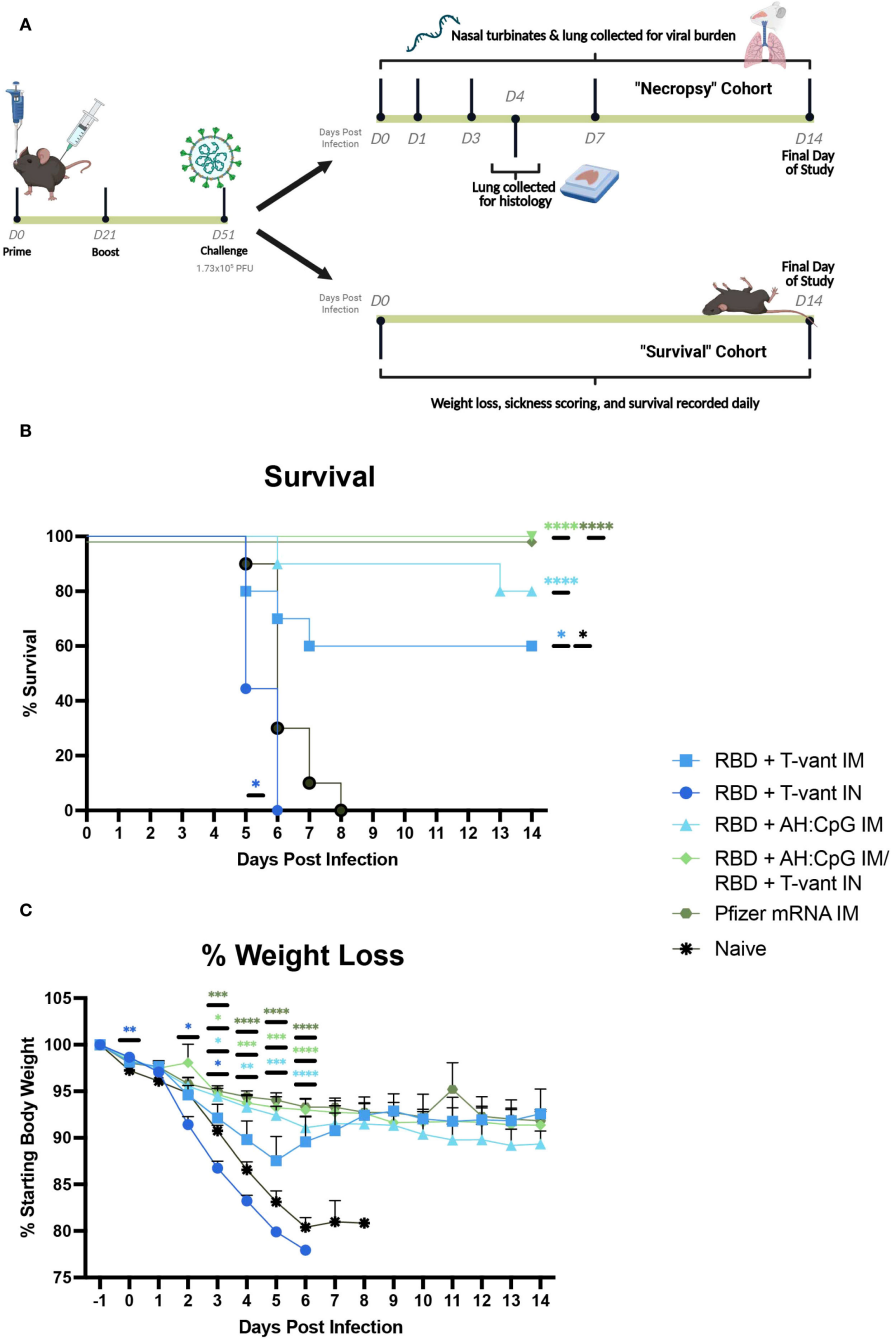
Route-specific protective efficacy of XBB.1.5 RBD vaccines. **(A)** Male and female B6.Cg-Tg(K18-ACE2)2Prlmn/J (K18-hACE2) mice were primed on day 0, then boosted on day 21. 30 days post boost (study day 51), mice were then challenged with 1.73x10^5^ PFU of SARS-CoV-2-XBB.1.5. Vaccines were administered either IM or IN. In the survival cohort of mice, animals were monitored and weighed daily. In the necropsy cohort, mice were sacrificed on days 0, 1, 3, 7, and 14 post-infection and nasopharynx and lung were collected and stored in Trizol. On day 4 post-infection, lungs were collected, inflated, and stored in Z-FIX fixative. All remaining animals were euthanized on day 14 post-infection. Figure created in BioRender. Hirsch, A. (2025) https://BioRender.com/0qriemf. **(B)** Survival analysis and **(C)** weight loss/gain (percentage of initial weight) (mean±s.e.m.) of 20- to 22-week-old male (n=5/group) and female (n=5/group) K18-hACE2 mice infected IN with 1.73x10^5^ PFU of SARS-CoV-2-XBB.1.5. Statistical significance was calculated by means of a **(B)** log-rank Mantel-Cox test and a **(C)** modified Chi-squared based method. Colored significance stars compare percent weight loss of vaccinated versus naive mice and black significance stars compare RBD + T-vant IM to RBD + AH: CpG IM/RBD + T-vant IN-immunized and mRNA-immunized mice. ^ns^ *p > 0.05*, *p ≤ 0.05, **p ≤ 0.01, ***p ≤ 0.001, or ****p ≤ 0.0001.

Current SARS-CoV-2 vaccines elicit limited cross-protective immunity against antigenically distant escape variants of concern (VOC), necessitating updated formulations to maintain or broaden protective efficacy (42–44). To date, few candidate vaccines targeting the Omicron-derived subvariant XBB.1.5 have been developed, and even fewer studies have assessed their protective efficacy (45, 46). In this study, we evaluated the protective efficacy of XBB.1.5 RBD subunit vaccines formulated with parenteral and mucosal adjuvants. These included (1) RBD + T-vant given IN; (2) RBD + T-vant given IM; (3) RBD + AH: CpG given IM; and (4) a heterologous “prime-pull” strategy combining RBD + AH: CpG IM and boosting with RBD + T-vant IN (**Table 1**). As a positive control, another group of mice received Omicron XBB.1.5 subvariant mRNA vaccine (Comirnaty; BioNTech-Pfizer, hereafter “mRNA IM”). K18-hACE2 mice were vaccinated on days 0 and 21, followed by intranasal challenge with 1.7x10^5^ PFU of SARS-CoV-2-XBB.1.5 one month later. This was selected based on a pilot study that demonstrated a dose of 1.7x10^5^ PFU was 100% lethal to naïve, aged (26- to 28-week) mice (**Supplementary Figure S1**). Mice were monitored for signs of terminal illness and weighed daily for 14 days post-infection (**Figure 1A**).

**Table 1:** Vaccine strategy and dosing for SARS-CoV-2 XBB.1.5 vaccine challenge study.

Vaccine strategy	RBD	T-vant	Alum	CpG-55.2	Route	Total volume delivered
RBD + T-vant IN	7 µg	5 µg			IN	10 µl
RBD + T-vant IM	7 µg	0.5 µg			IM	50 µl
RBD + AH: CpG IM	7 µg		200 µg	10 µg	IM	50 µl
RBD + AH: CpG IM Prime/RBD + T-vant IN Boost	7 ug	5 ug	200 µg	10 µg	IM/IN	50 µl/10 µl
Pfizer mRNA IM	COMIRNATY Omicron XBB.1.5: diluted 1:3 in PBS; 1.67 µg per dose				IM	50 µl
T-vant Only IN		5 µg			IN	10 µl
T-vant Only IM		0.5 µg			IM	50 µl
AH: CpG Only IM			200 µg	10 µg	IM	50 µl
AH: CpG Only IM Prime/T-vant Only IN Boost		5 µg	200 µg	10 µg	IM/IN	50 µl/10 µl
RBD Only IM	7 µg				IM	50 µl
Naive						

The prime-pull immunization conferred complete protection against an otherwise lethal XBB1.5 challenge, similar to the protection achieved with the mRNA IM vaccine (p ≤ 0.0001 compared to naïve; **Figure 1C**). Mice immunized with RBD + AH: CpG IM or RBD + T-vant IM exhibited 80% and 60% survival, respectively, compared to ≤20% survival in vaccine control groups (p ≤ 0.0001 and p ≤ 0.05 compared to naive, respectively; **Figure 1B** **;** **Supplementary Figure S2**). Mice immunized with RBD + T-vant IN showed accelerated disease progression compared to naïve mice, with complete mortality by 6 DPI (p ≤ 0.05; **Figure 1C**). Weight loss mirrored survivability outcomes. RBD + AH: CpG, RBD + AH: CpG IM/RBD + T-vant IN, and mRNA IM-immunized mice demonstrated reduced weight loss compared to naïve mice, while RBD + T-vant IN-immunized mice experienced exacerbated weight loss compared to naïve mice (**Figure 1C**).

### Accelerated SARS-CoV-2 clearance via mRNA, RBD + AH: CpG IM, and prime-pull vaccination

Next-generation mucosal vaccines must promote viral clearance from the respiratory tract to reduce both viral replication in the host and transmission between individuals (20). Therefore, we monitored viral burdens in the nasopharynx and lungs of immunized mice (aged 6 to 8 weeks old) following challenge with a sub-lethal dose of XBB1.5 (1.7x10^5^ PFU). The experimental detail is provided in **Table 1**. Lung and nasopharynx tissues were harvested at 1, 3, and 7 DPI (**Figure 1A**) and RT-qPCR was used to measure the abundance of subgenomic RNA for the N protein of SARS-CoV-2.

**Figure 2:**
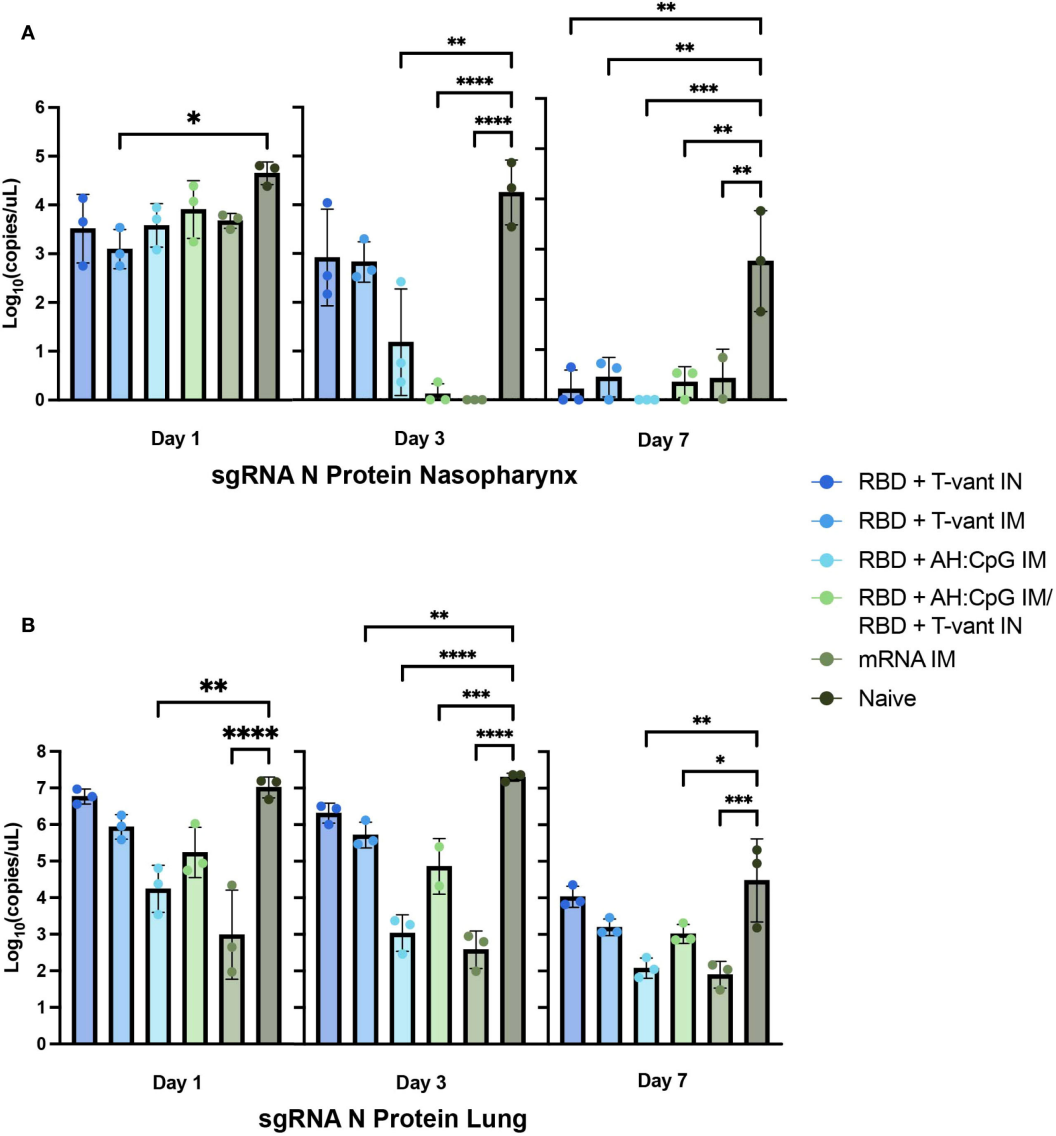
Accelerated respiratory SARS-CoV-2 clearance via mRNA, RBD + AH: CpG IM, and prime-pull vaccination. Six- to eight-week-old male and female (n=3/group) K18-hACE2 mice were immunized and then challenged with SARS-CoV-2-XBB.1.5. At 1, 3, and 7 days post infection, mice were euthanized and RNA was isolated from **(A)** nasopharynx and **(B)** lung tissues. Subgenomic RNA N viral copies were quantified in terms of log_10_(copies/µL). Statistical significance was calculated by means of a one-way ANOVA followed by *post hoc* Tukey’s test for multiple comparisons. Significance bars indicate significantly different populations. ^ns^ *p > 0.05*, *p ≤ 0.05, **p ≤ 0.01, ***p ≤ 0.001, or ****p ≤ 0.0001.

In the nasopharynx at 1 DPI, only mice immunized with RBD + T-vant IM displayed a significant reduction in viral burden compared to naïve mice (p ≤ 0.05). However, by 3 DPI, the RBD + AH: CpG IM (p ≤ 0.01), RBD + AH: CpG IM/RBD + T-vant IN (p ≤ 0.0001), and mRNA IM (p ≤ 0.0001)-immunized mice displayed significantly lower viral loads compared to naïve mice, with little to no virus detected in mice receiving RBD + AH: CpG IM/RBD + T-vant IN and mRNA IM vaccines (**Figure 2A**), suggesting that these regimens had decreased SARS-CoV-2 in the nasopharynx at critical early timepoints. By 7 DPI, all vaccinated groups exhibited significantly lower viral burdens compared to naïve mice (**Figure 2A**).

In the lung at 1 DPI, mice immunized with RBD + AH: CpG IM (p ≤ 0.01) or mRNA IM (p ≤ 0.0001) showed a significant reduction in viral burden compared to naïve mice (**Figure 2B**). By 3 DPI, all vaccinated groups, with the exception of RBD +T-vant IN-immunized mice, demonstrated significantly lower lung viral loads compared to naïve mice; however, the lowest burdens were observed in the RBD + AH: CpG IM and mRNA IM-immunized mice (**Figure 2B**). At 7 DPI, mice immunized with RBD + AH: CpG IM (p ≤ 0.01), mRNA IM (p ≤ 0.001), or RBD + AH: CpG IM/RBD + T-vant IN (p ≤ 0.05) displayed significantly reduced viral burdens, compared to naïve mice (**Figure 2B**). Overall, lung data indicate that the RBD + AH: CpG IM and mRNA IM vaccines were most effective at promoting early viral clearance in the lung.

### Histopathological analysis of vaccine protection against lung pathology

**Figure 3:**
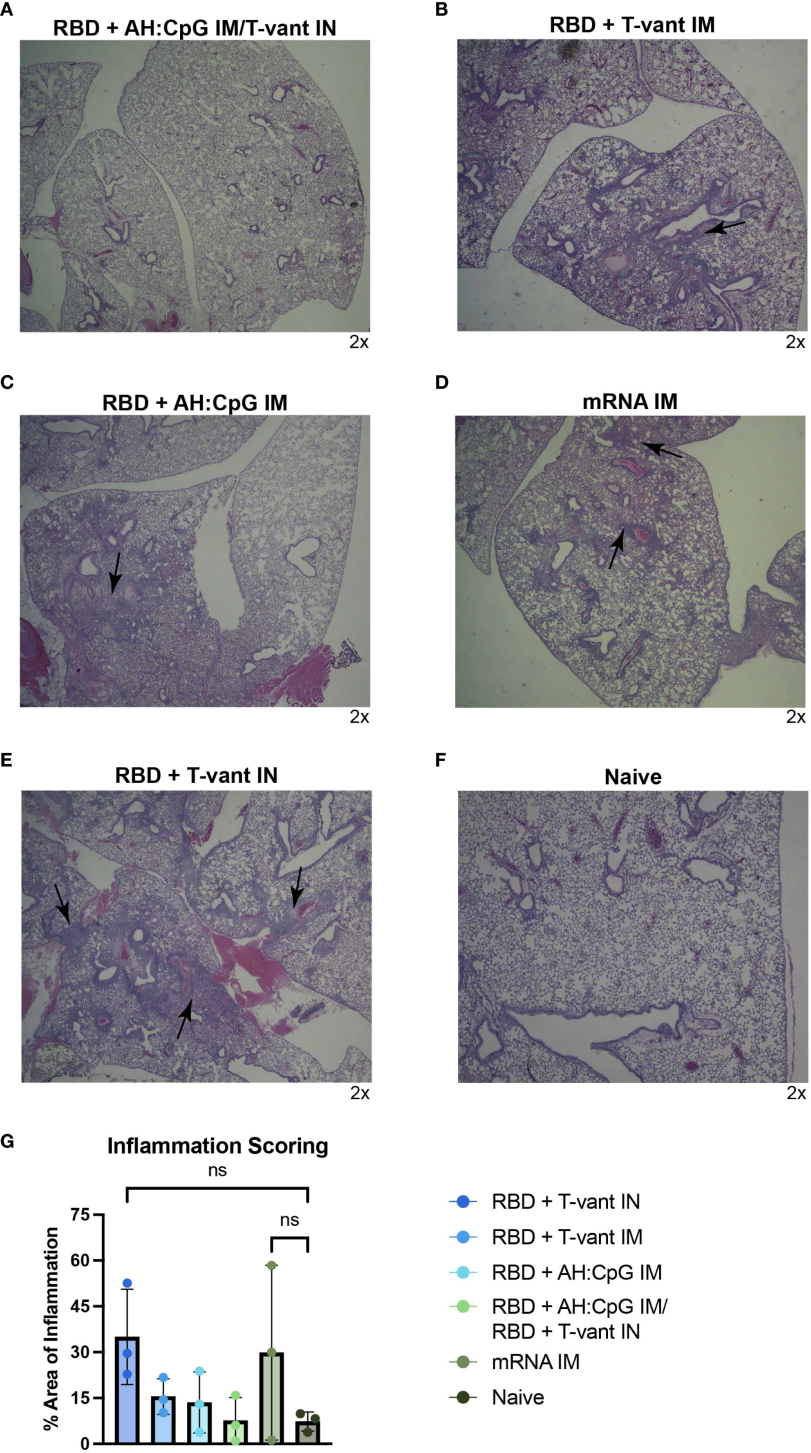
Histopathological analysis reveals route-dependent protection against lung pathology. Six- to eight-week-old male and female (n=3/group) K18-hACE2 mice were immunized then challenged with SARS-CoV-2-XBB.1.5. H&E staining of lung sections is shown. **(A-F)** Images show low-power (2x) magnifications. H&E staining results are representative of multiple sections from three mice per group. Arrows indicate areas of inflammation. **(G)** Quantification of lung inflammation by percentage of the lung affected at 4 days post infection. Statistical significance was calculated by means of a one-way ANOVA followed by *post hoc* Tukey’s test for multiple comparisons. ^ns^ *p > 0.05*.

Histopathological analyses were performed on lung tissue harvested from vaccinated mice 4 DPI with SARS-CoV-2-XBB.1.5. Lungs were fixed, sectioned, and stained with H&E to assess inflammation, cell infiltration, and overall pathology. Whole-slide images were then created via digital scan by a Zeiss Axio Scan.Z1, and analyzed with computer software (HALO v3.1, Indica Labs). Immunization with RBD + AH: CpG/RBD + T-vant IN appeared to protect mice from any lung pathology (**Figure 3A**). In contrast, RBD + T-vant IN immunization enhanced lung pathology, with marked inflammation and vasculitis in all mice (p=0.086 compared to naïve; **Figure 3E**). There was some generalized inflammation and vasculitis observed in mice immunized with RBD + T-vant IM (**Figure 3B**) and RBD + AH: CpG IM (**Figure 3C**), and mRNA (**Figure 3D**) however the overall scoring was not statistically different among any of the groups (**Figure 3G**).

### AH: CpG and T-vant adjuvanted RBD vaccines drive Th1 and Th17 immunity, respectively

T cells play a critical protective role against COVID-19 through viral clearance, disease mitigation, complementation of antibody responses, and cross-reactive immunity across viral variants (47–50). Respiratory mucosal T helper type (Th) 1 immune polarization is particularly vital for successful antiviral defenses, whereas Th2/Th17 biases correlate with severe COVID-19 disease in humans (51–53). To evaluate vaccine-elicited T cell immunity, mice were immunized with XBB.1.5 RBD vaccines (as outlined in **Table 1**) and tissues were harvested 2 weeks post final immunization. Mucosal and systemic effector T cell cytokine profiles were assessed in the lung and spleen via *ex vivo* RBD restimulation, intracellular cytokine staining, and flow cytometry. The percentage of antigen-specific T cells producing IFN-γ (Th1), IL-4 (Th2), and IL-17 (Th17) was determined using the gating strategy shown in **Supplementary Figure S3**.

**Figure 4:**
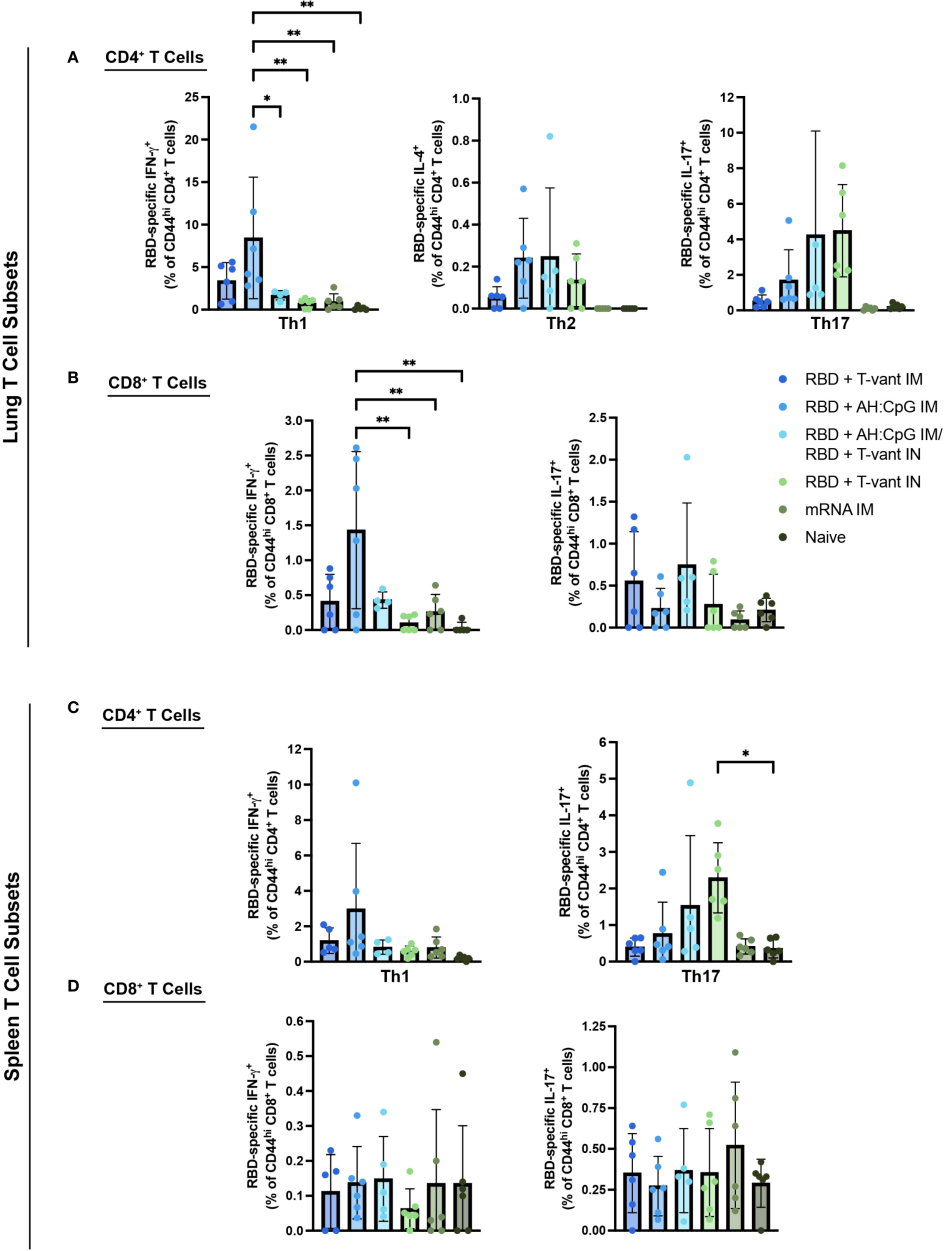
AH: CpG and T-vant adjuvanted RBD vaccines drive Th1 and Th17 immunity, respectively. Single cell suspensions were prepared from lung and spleen tissues of mice (n=5-6/group) and restimulated *ex vivo* with XBB.1.5 RBD antigen. Intracellular staining for IFN-γ (APC), IL-4 (BV605), and IL-17 (PE) was performed, and cells were gated on **(A)** lung CD4^+^, CD44^hi^, **(B)** lung CD8^+^, CD44^hi^, **(C)** spleen CD4^+^, CD44^hi^, or **(D)** spleen CD8^+^, CD44^hi^ populations by flow cytometry. Statistical significance was calculated by means of a one-way ANOVA followed by *post hoc* Tukey’s test for multiple comparisons. Significance bars indicate significantly different populations. ^ns^ *p > 0.05*, *p ≤ 0.05, or **p ≤ 0.01.

Homologous RBD + AH: CpG IM immunization induced significantly more IFN-γ-producing CD4^+^ in the lung compared to all other groups with the exception of RBD + T-vant IM (**Figure 4A**). There were no significant increases in IL-4- or IL-17-producing CD4^+^ T cells in the lungs of mice for any vaccine group compared to naïve mice (**Figure 4A**). RBD + AH: CpG IM immunization also promoted significantly more IFN-γ-producing CD8^+^ T cells in the lung compared to all groups except for RBD + T-vant IM and the RBD + AH: CpG/RBD + T-vant IN groups (**Figure 4B**). In the spleen, IFN-γ-producing CD4^+^ T cells were higher in RBD + AH: CpG IM-immunized mice, though the response was not statistically significant compared to naïve mice (p=0.067; **Figure 4C**). Homologous RBD + T-vant IN immunization induced significantly more IL-17-producing CD4^+^ T cells in the spleen compared to naïve mice (p ≤ 0.05; **Figure 4C**). There was no difference in IFN-γ- or IL-17- producing CD8^+^ T cells in the spleen for any of the vaccine groups (**Figure 4D**).

### Anti-RBD serum IgG and neutralizing antibody responses are elevated in intramuscularly immunized mice

**Figure 5:**
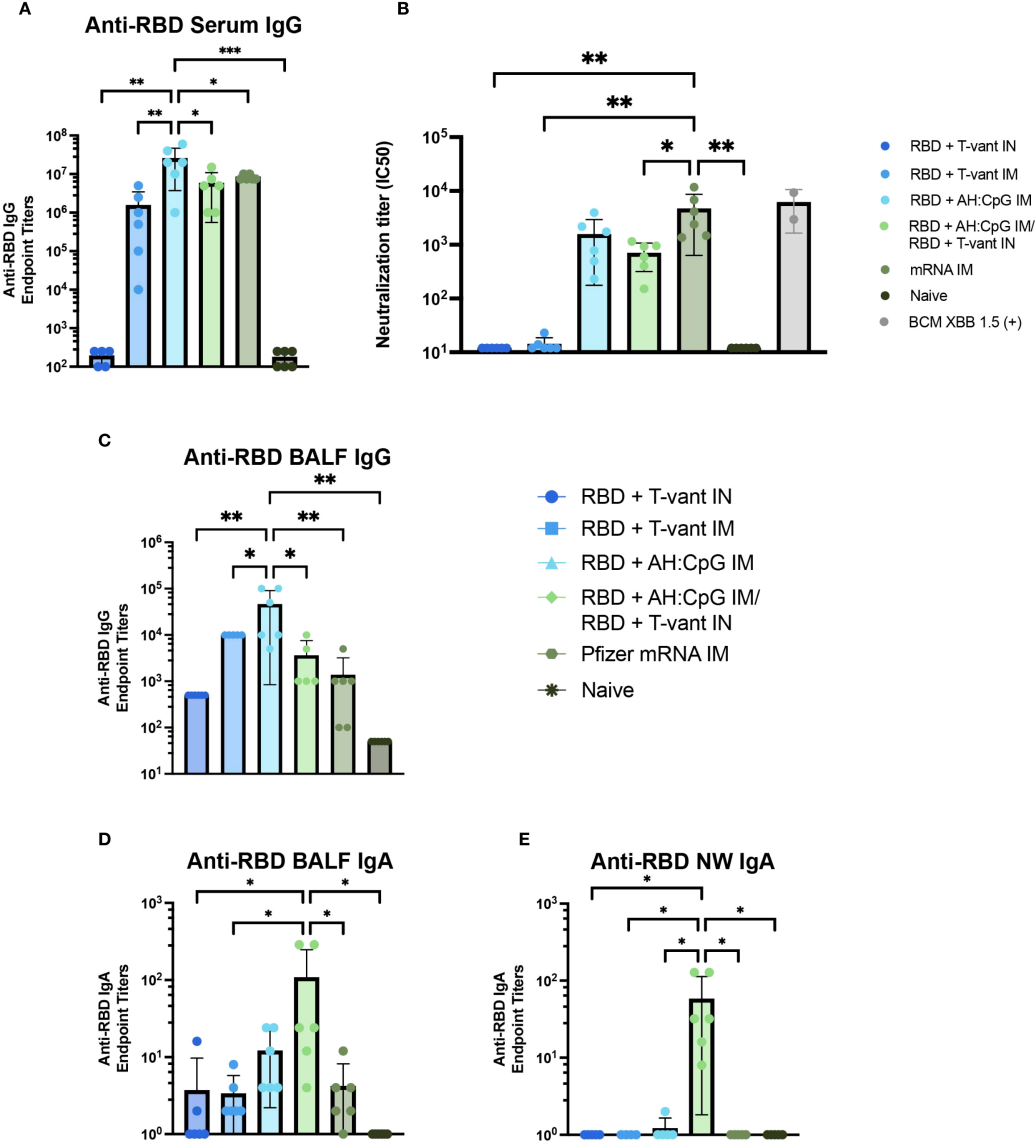
Mucosal boosting with T-vant adjuvant elicits robust mucosal IgA, systemic IgG, and robust neutralizing antibodies. Sera, BALF, and NW were obtained two weeks post-boost (study day 35) (n=6/group). XBB.1.5 RBD-specific reciprocal endpoint titers were measured via indirect ELISA in the **(A)** serum, **(C, D)** BALF, and **(E)** NW. **(B)** Neutralizing antibody titers against SARS-CoV-2 XBB.1.5. Individual IC_50_ values (n=6 mice) are plotted on a log_10_ scale. An in-house generated control serum (BCM XBB 1.5) was included to the assay as a positive control (n=2). Statistical significance was calculated by means of a one-way ANOVA followed by *post hoc* **(A, C-E)** Tukey’s test or **(B)** Holm-Sidak’s test for multiple comparisons. Significance bars indicate significantly different populations. ^ns^ *p > 0.05*, *p ≤ 0.05, **p ≤ 0.01, or ***p ≤ 0.001.

Robust serum antibody responses are critical for SARS-CoV-2 vaccine-mediated protection (54, 55). We first assessed anti-XBB.1.5 RBD IgG titers in the serum of immunized mice. Homologous RBD + AH: CpG IM-immunized mice generated the highest serum IgG compared to all other vaccine strategies (**Figure 5A**), consistent with prior studies demonstrating the enhanced capacity of AH: CpG-adjuvanted vaccines to induce antigen-specific serum IgG in SARS-CoV-2 vaccines (27, 28).

Vaccine-induced neutralizing antibody is essential to block viral entry into cells and thereby achieve sterilizing immunity (56, 57) – a key objective of next-generation vaccines (56). Therefore, a pseudovirus neutralization assay was used to assess the neutralizing capacity of serum antibodies elicited by immunization. Mice immunized with mRNA IM exhibited significantly higher neutralizing titers than all other vaccine strategies, with the exception of the RBD + AH: CpG IM group (**Figure 5B**). Notably, there was no significant difference in neutralizing capacity between mice immunized with RBD + AH: CpG IM and RBD + AH: CpG IM/RBD + T-vant IN (**Figure 5B**), suggesting that the substitution of a mucosal boost did not compromise the induction of neutralizing antibodies against the XBB.1.5 variant of SARS-CoV-2.

### Heterologous prime-pull vaccination promotes robust IgA responses in the respiratory tract

Mucosal antibodies, secretory IgA in particular, are essential in limiting SARS-CoV-2 infection and transmission (54, 55). Similar to what was observed in the serum, homologous RBD + AH: CpG IM-immunized mice produced higher titers of anti-RBD IgG in the BALF than all other vaccine groups (**Figure 5C**). In contrast, the RBD + AH: CpG/RBD + T-vant IN-immunized mice exhibited significantly higher BALF IgA compared to naïve, mRNA IM, RBD + T-vant IM, and RBD + T-vant IN immunized groups (p ≤ 0.05; **Figure 5D**). In the nasal wash, mice immunized with RBD + AH: CpG IM/RBD + T-vant IN displayed the highest titers of anti-XBB.1.5 RBD IgA compared to all other vaccine groups (p ≤ 0.05; **Figure 5E**).

## Discussion

Here, we evaluated the protective efficacy of multiple SARS-CoV-2 vaccination regimens, including homologous prime-boost and heterologous prime-pull strategies. We demonstrated that prime-pull mucosal immunization with an adjuvanted RBD subunit vaccine provided equivalent protection to mRNA vaccination against SARS-CoV-2 challenge in aged mice. This result is significant for two reasons: 1) current SARS-CoV-2 mRNA vaccines have demonstrated remarkable efficacy in preventing severe COVID-19 (58–60) and 2) the prime-pull vaccination strategy successfully protected aged mice – a population known to experience diminished vaccine efficacy (61, 62). SARS-CoV-2-XBB.1.5 variant infection in mice mirrors disease in humans, where severity and mortality predominantly occur in older subjects (45, 63, 64). Consistent with the survival outcomes in aged mice, similar results were observed in immunized young mice where both prime-pull and mRNA immunization effectively cleared the virus by 3 days post infection in the upper respiratory tract. Interestingly, the prime-pull strategy appeared to completely mitigate lung pathology in mice however we were unable to demonstrate statistical differences compared to other vaccine groups due to the small sample sizes.

From a humoral immunity perspective, parenteral immunization in our study generated higher titers of antigen-specific serum IgG compared to intranasal immunizations. However, the prime-pull approach induced antigen-specific IgA in the nasal cavity at titers significantly higher than those achieved by parenteral vaccination. The presence of increased mucosal IgA may be a key driver of the enhanced protection observed in prime-pull-vaccinated mice, particularly in reducing mortality, viral load, and lung pathology. Additionally, mucosal IgA may play a critical role in limiting viral shedding and transmission, as demonstrated in prior studies on SARS-CoV-2 and influenza (65–69). Regarding antibody function, only AH: CpG-adjuvanted RBD (both homologous and prime-pull immunizations) and mRNA vaccines exhibited serum neutralizing antibodies, as assessed by pseudovirus assay. It is possible that the prime-pull immunization promoted neutralizing antibodies in the mucosa, however, this was not evaluated in our study. It is also possible that prime-pull vaccination promoted the recruitment of systemic antibodies or T cells, induced by the parenteral prime, to the respiratory mucosa via the mucosal boost. Future studies will focus on identifying the specific mechanisms of protection for the prime-pull immunization strategy. Additionally, while our study focused on the XBB.1.5 subvariant, the cross-protective efficacy against other variants of the prime-pull regimen remains unknown and warrants further evaluation. Expanding the application of this prime-pull regimen to other respiratory pathogens is another important avenue for future research. Given its success against SARS-CoV-2, the heterologous intramuscular prime with AH: CpG and intranasal boost with T-vant adjuvants may serve as a broadly applicable strategy for mucosal immunization against diverse respiratory threats. Potentially, this approach could augment immunization strategies aimed not only at reducing community morbidity and mortality, but also as a means to slow or halt community-based virus transmission.

Despite using the same mucosal adjuvant, the homologous intranasal immunization with RBD + T-vant resulted in starkly different outcomes compared to the prime-pull regimen and led to exacerbation of morbidity, mortality, and lung tissue inflammation. This raises critical questions regarding the underlying immunological mechanisms driving this disparity. Parenteral vaccines adjuvanted with AH: CpG primarily induce a Th1-skewed immune response, a well-established correlate of protection against SARS-CoV-2 (50, 70). One potential explanation for the detrimental outcome following homologous intranasal immunization is the known induction of a Th17-dominant immune response after intranasal immunization, regardless of the vaccine adjuvant (71). Although we did not observe increased numbers of lung IL-17-producing CD4^+^ T cells in RBD + T-vant IN-immunized mice, there was a Th17 polarization observed in the spleen. A similar Th17 bias was reported with an intranasal subunit vaccine adjuvanted with CRX-601 (a synthetic TLR-4 agonist), where vaccine-induced Th17 responses exacerbated morbidity following influenza infection (72). It is possible that excessive Th17-driven inflammation may underlie the deleterious effects observed with homologous intranasal T-vant vaccination against SARS-CoV-2, indicating that multiple doses or an intranasal prime may be detrimental in this scenario. This view is consistent with our previous hypothesized role of Th17 responses underlying coronavirus immunopathology and immune enhancement (73). Notably, a single booster dose containing T-vant in the prime-pull regimen was beneficial and T-vant itself is not inherently damaging when used in intranasal vaccines. Prior studies have demonstrated that mucosal Th17 responses mediate protection against certain respiratory pathogens, such as *Bordetella pertussis* (36). Furthermore, T-vant adjuvanted RBD vaccine given intramuscularly provided 60% protection against XBB1.5 in aged mice. Our findings highlight the need to carefully evaluate the safety and protective efficacy of mucosal vaccines in relevant challenge models, particularly for respiratory pathogens.

In conclusion, prior studies have demonstrated that prime-pull mucosal vaccination can enhance protection against mucosal pathogens (54, 74). Our results further establish T-vant as a potent mucosal adjuvant within this context. Importantly, given that much of the global population has already received parenteral SARS-CoV-2 immunization or has been exposed to the virus naturally, the prime-pull strategy presents a translational advantage, leveraging pre-existing systemic immunity with a single mucosal boost.

## Data Availability

The original contributions presented in the study are included in the article/**Supplementary Material**. Further inquiries can be directed to the corresponding author.

## Supplementary material

Supplementary Figure 1 1.73x10^5^ PFU of SARS-CoV-2-XBB.1.5 achieves lethality in aged mice. (**A**) 26- to 28-week old (n=3/group) female B6.Cg-Tg(K18-ACE2)2Prlmn/J (K18-hACE2) mice were infected with 0 PFU, 1.73x10^3^ PFU, 1.73x10^4^ PFU, or 1.73x10^5^ PFU of SARS-CoV-2-XBB.1.5. Mice were monitored and weighed daily until 14 DPI. Figure created with BioRender.com. (**B**) Survival analysis and (**C**) weight loss/gain (percentage of initial weight) (mean±s.e.m.) of 26- to 28-week-old K18-hACE2 mice infected intranasally with an increasing dose of SARS-CoV-2. Colored significance stars compare infected versus sham mice; black significance stars compare 1.72x10^3^ PFU and 1.72x10^5^ PFU doses. Statistical significance was calculated by means of a (B) log-rank Mantel-Cox test and a (**C**) modified Chi-squared based method; ^ns^ *p > 0.05*, **p ≤ 0.05*, ***p ≤ 0.01*, ****p ≤ 0.001*, or *****p ≤ 0.0001*.

Supplementary Figure 2 Protective efficacy of RBD vaccine control groups. Survival analysis of 20- to 22-week-old male (n=5/group) and female (n=5/group) K18-hACE2 mice infected intranasally with 1.73x10^5^ PFU of SARS-CoV-2-XBB.1.5.

Supplementary Figure 3 Gating strategy for intracellular cytokine flow cytometry analysis. Initially, a sub-gate of single cells was identified. Dead and CD45^-^ cells were then excluded by gating on Zombie NIR^-^ and CD45^+^ populations, respectively. Lymphocytes were identified by gating on side scatter vs forward scatter populations. CD3^+^, Dump^-^ cells were sub-gated, then CD4^+^ and CD8^+^ cells were identified. Subsequently, individual intracellular staining for IFN-γ, IL-4, or IL-17 was plotted against CD44^hi^ T cell populations. Percent of parent populations were reported.
