# Supplementary figures and images for: Heterologous prime-pull mucosal vaccination with an adjuvanted RBD vaccine elicits robust IgA production and protects against SARS-CoV-2
Source: Front Immunol. 2025 Sep 19;16:1673460. doi: 10.3389/fimmu.2025.1673460 (PMC12491188; doi:10.3389/fimmu.2025.1673460)

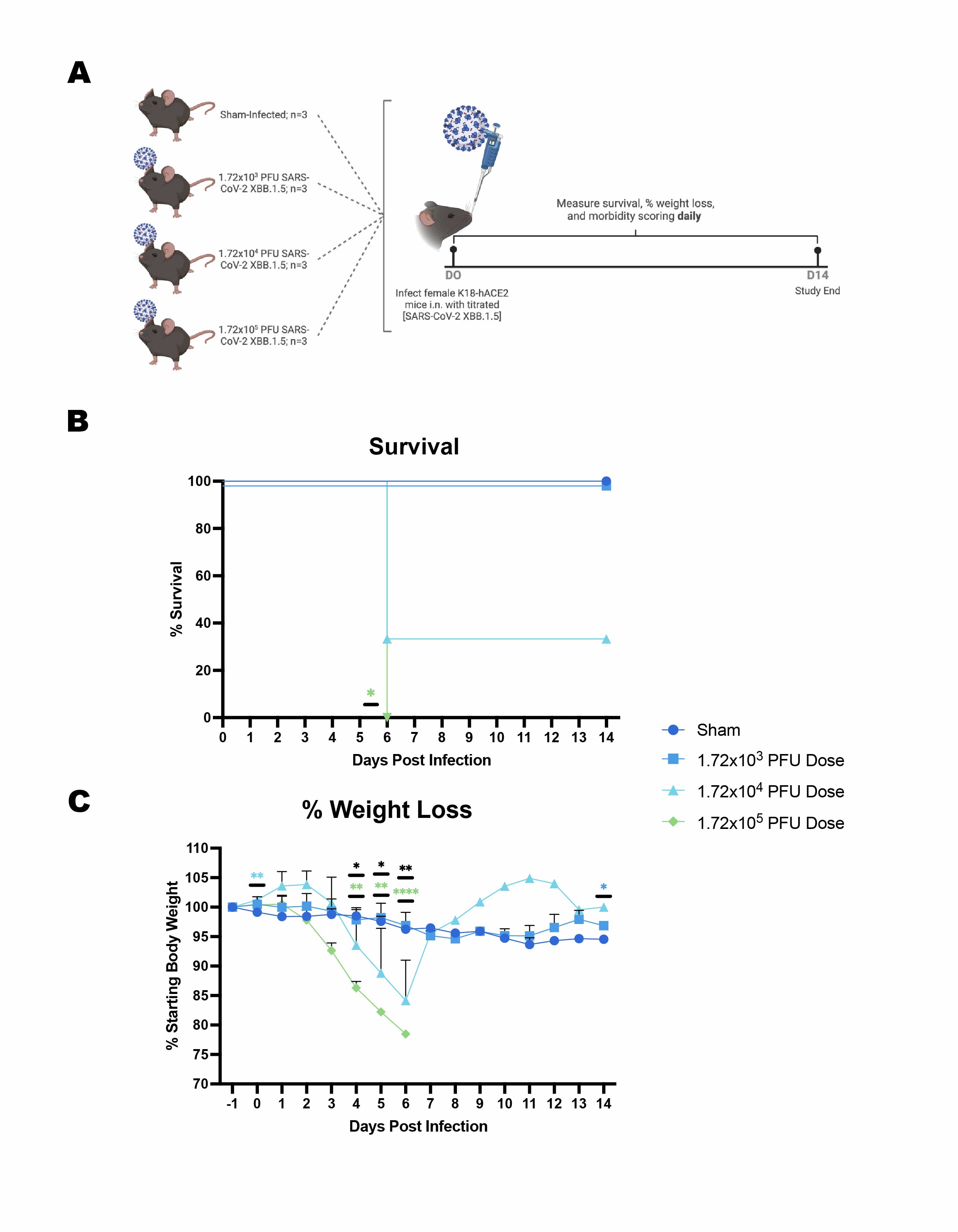

Supplement: Supplementary Figure 1 — 1.73x10^5^ PFU of SARS-CoV-2-XBB.1.5 achieves lethality in aged mice. (A) 26- to 28-week old (n=3/group) female B6.Cg-Tg(K18-ACE2)2Prlmn/J (K18-hACE2) mice were infected with 0 PFU, 1.73x10^3^ PFU, 1.73x10^4^ PFU, or 1.73x10^5^ PFU of SARS-CoV-2-XBB.1.5. Mice were monitored and weighed daily until 14 DPI. Figure created with BioRender.com. (B) Survival analysis and (C) weight loss/gain (percentage of initial weight) (mean±s.e.m.) of 26- to 28-week-old K18-hACE2 mice infected intranasally with an increasing dose of SARS-CoV-2. Colored significance stars compare infected versus sham mice; black significance stars compare 1.72x10^3^ PFU and 1.72x10^5^ PFU doses. Statistical significance was calculated by means of a (B) log-rank Mantel-Cox test and a (C) modified Chi-squared based method; ns p > 0.05, *p ≤ 0.05, **p ≤ 0.01, ***p ≤ 0.001, or ****p ≤ 0.0001. [file Image1.jpeg]

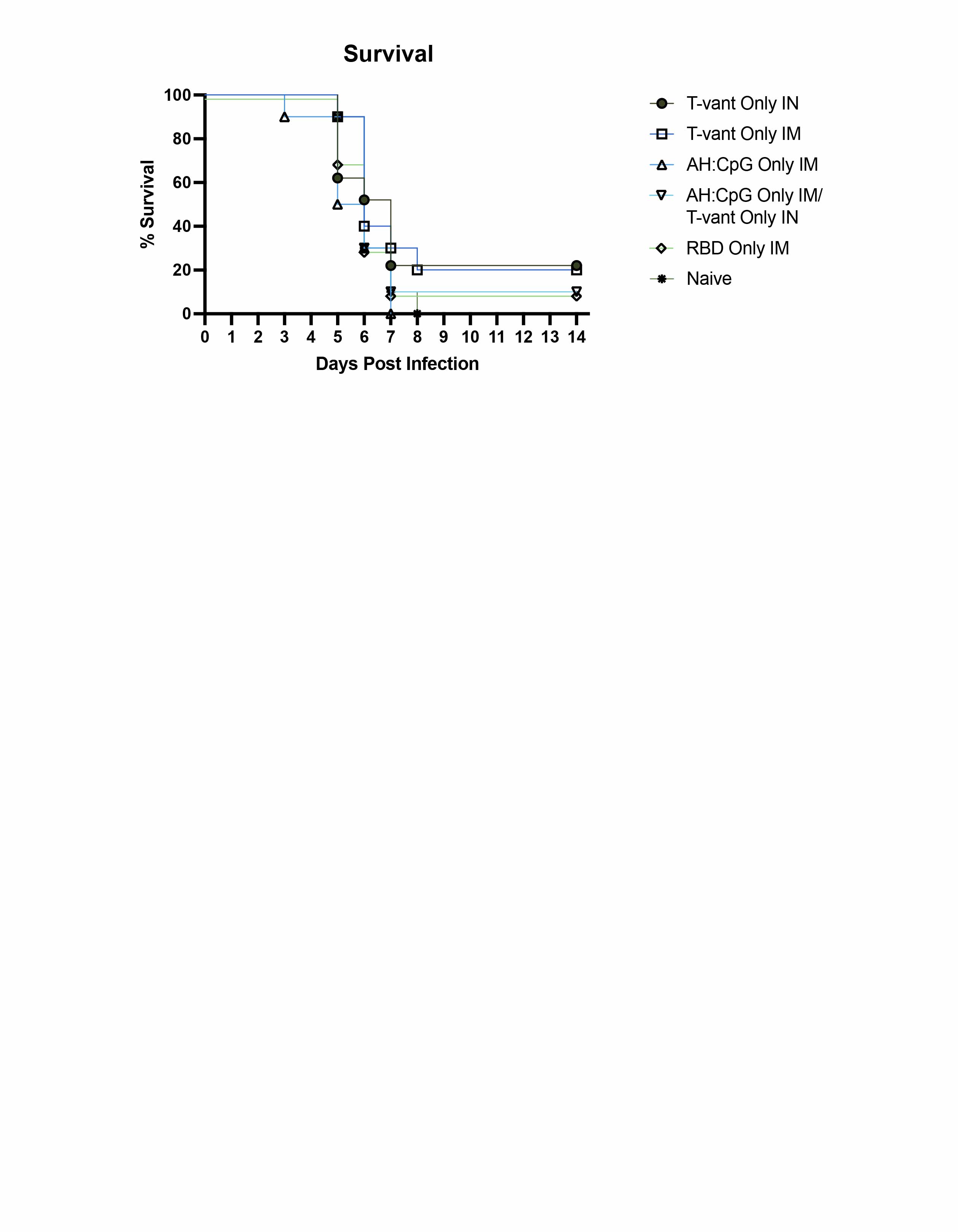

Supplement: Supplementary Figure 2 — Protective efficacy of RBD vaccine control groups. Survival analysis of 20- to 22-week-old male (n=5/group) and female (n=5/group) K18-hACE2 mice infected intranasally with 1.73x10^5^ PFU of SARS-CoV-2-XBB.1.5. [file Image2.jpeg]

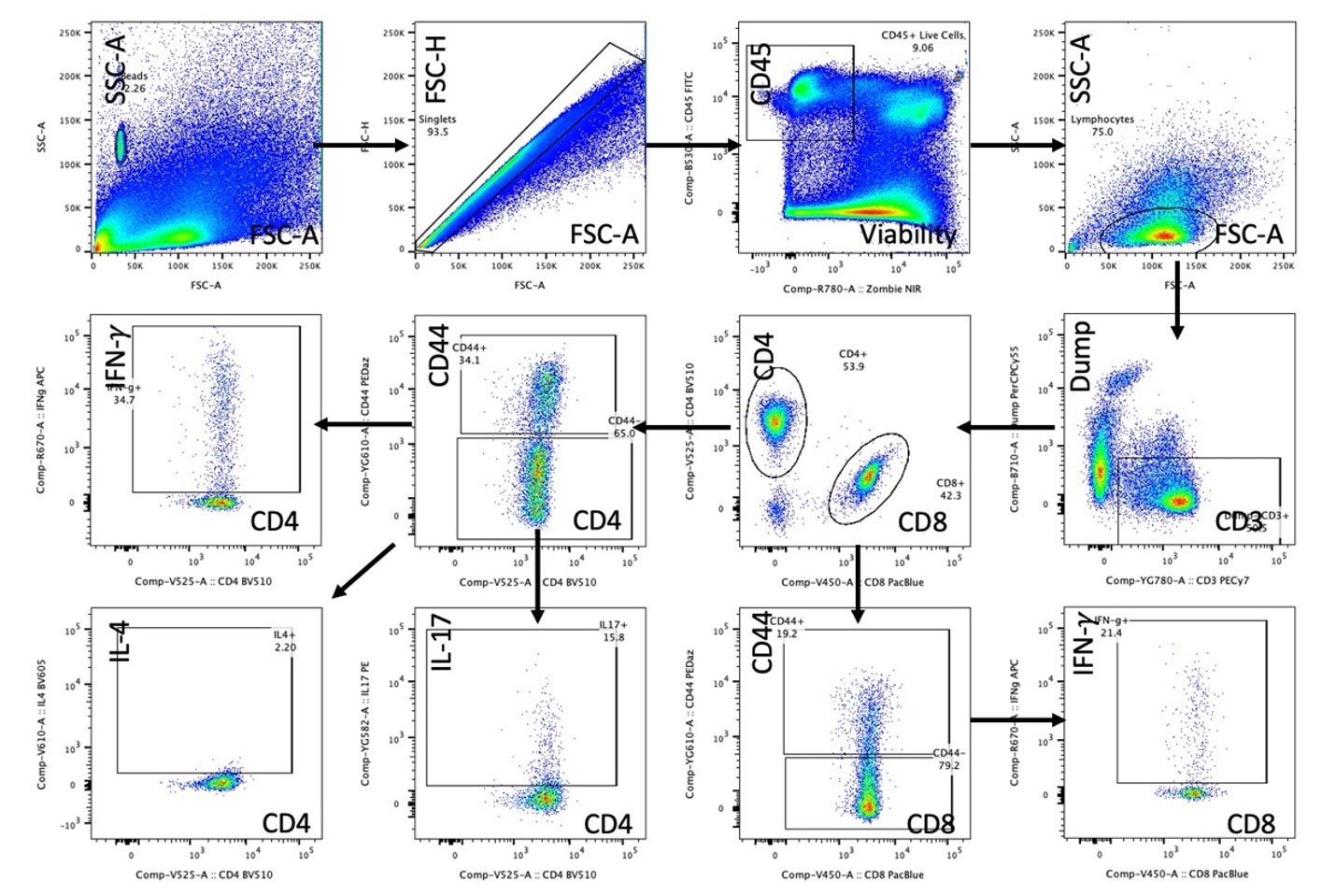

Supplement: Supplementary Figure 3 — Gating strategy for intracellular cytokine flow cytometry analysis. Initially, a sub-gate of single cells was identified. Dead and CD45- cells were then excluded by gating on Zombie NIR- and CD45+ populations, respectively. Lymphocytes were identified by gating on side scatter vs forward scatter populations. CD3+, Dump- cells were sub-gated, then CD4+ and CD8+ cells were identified. Subsequently, individual intracellular staining for IFN-γ, IL-4, or IL-17 was plotted against CD44hi T cell populations. Percent of parent populations were reported. [file Image3.jpeg]
